# Delayed Surgical Management of Acute Type A Aortic Dissection in a Patient with Recent COVID-19 Infection and Post-COVID-19 Bronchopneumonia—Case Report and Review of Literature

**DOI:** 10.3390/medicina58101357

**Published:** 2022-09-27

**Authors:** Mircea Robu, Diana Romina Marian, Rasvan Vasile, Bogdan Radulescu, Alice Stegaru, Cristian Voica, Claudia Nica, Daniela Gheorghita, Ondin Zaharia, Antoniac Iulian, Angelica Moldovan, Victor Pavel, Horatiu Moldovan, Vlad Anton Iliescu

**Affiliations:** 1 Department of Cardiovascular Surgery, Prof. Dr. C.C. Iliescu Emergency Institute for Cardiovascular Diseases, 022322 Bucharest, Romania; 2 Anesthesia and Intensive Care Department, Prof. Dr. C.C. Iliescu Emergency Institute for Cardiovascular Diseases, 022328 Bucharest, Romania; 3 Department of Cardiovascular Surgery, Clinical Emergency Hospital Bucharest, 014461 Bucharest, Romania; 4 Faculty of Materials Science and Engineering, Politehnica University of Bucharest, 060042 Bucharest, Romania; 5 Faculty of Medicine, Carol Davila University of Medicine and Pharmacy, 050474 Bucharest, Romania; 6 Prof. Dr. Theodor Burghele Clinical Hospital, 050659 Bucharest, Romania; 7 Academy of Romanian Scientists, 54, Spl. Independentei, 050711 Bucharest, Romania

**Keywords:** type A aortic dissection, COVID-19, malperfusion syndrome, bronchopneumonia, respiratory failure, cardiopulmonary bypass

## Abstract

Ever since it was first described in 1760, acute type A aortic dissection has created difficulties in its management. The recent COVID-19 pandemic revealed that extrapulmonary manifestations of this condition may occur, and recent reports suggested that aortic dissection may be amongst them since it shares a common physiopathology, that is, hyper-inflammatory syndrome. Cardiac surgery with cardiopulmonary bypass in the setting of COVID-19-positive patients carries a high risk of postoperative respiratory failure. While the vast majority accept that management of type A aortic dissection requires urgent surgery and central aortic therapy, there are some reports that advocate for delaying surgery. In this situation, the risk of aortic rupture must be balanced with the possible benefits of delaying urgent surgery. We present a case of acute type A dissection with COVID-19-associated bronchopneumonia successfully managed after delaying surgery for 6 days.

## 1. Introduction

Aortic dissection (AD) was first described by Frank Nicholls, King George II of England’s personal physician. He described the classical findings of AD in the king’s autopsy in 1760. AD is the most catastrophic form of acute aortic syndrome, consisting of an intimo-medial tear that allows blood flow to enter the aortic wall and create a secondary lumen, most commonly referred to as false lumen, with severe consequences. Without treatment, the mortality is 1% per hour, with half of patients dead by the third day [1,2,3].

Respiratory infection with COVID-19 has reached a pandemic level. Although it is primarily an acute respiratory disease, extrapulmonary manifestations such as cardiovascular complications exist [4]. There have been reports that AD could be an extrapulmonary manifestation of this condition, considering that both share the same physiopathogenic mechanism [5,6,7,8,9].

Urgent surgery in patients with AD has a class I indication according to the ESC Guidelines on the diagnosis and treatment of aortic disease [10]. However, there are some that advocate for delaying surgery in certain situations because of a poor postoperative prognosis, such as in cases of an initial presentation with malperfusion syndrome or organ ischemia [11,12]. 

We present a case of type A aortic dissection complicated by COVID-19-associated bronchopneumonia and respiratory failure, successfully managed after delaying surgery for 6 days. 

## 2. Case Report

**Figure 1:**
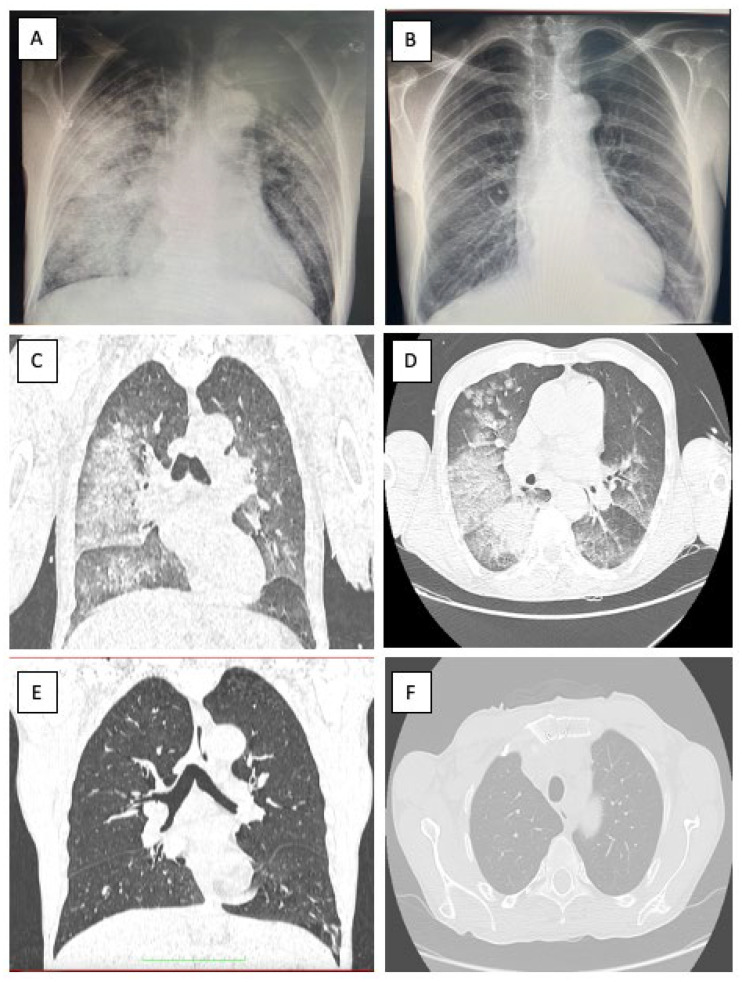
(**A**) Chest XR at presentation showing bilateral alveolar infiltrates, with both central and peripheric distribution in the inferior two-thirds of both lungs (more important on the **right**)—suggesting an infectious pulmonary disease; (**B**) chest XR at discharge showing resolution of pulmonary lesions; (**C**,**D**) computed tomography aspect of the lungs at presentation resembling multiple, bilateral areas of consolidation, with air bronchogram included and areas of ground-glass attenuation with a mainly central distribution, in the two-thirds of both pulmonary parenchyma (more significant in the right lung) suggesting an infectious pneumonia; (**E**,**F**) computed tomography aspect of the lungs at discharge with resolution of initial lesions.

A 51-year-old male, with no cardiovascular risk factors, was referred to our institution from a tertiary center for type A aortic dissection (TAAD) diagnosed using chest computed tomography (CT). His medical history revealed a syncopal episode a day before admission and a recent respiratory infection approximately 2 weeks before admission managed outside the hospital with antibiotherapy stopped by the patient after 2 days. The patient was not vaccinated for COVID-19. At admission, the patient was febrile and uncooperative with psychomotor agitation. The patient was spontaneously breathing and presented a productive cough; pulse oximetry revealed a 91% oxygen saturation with 10 L of oxygen from the facial mask. The patient was stable, his systolic blood pressure was 100–120 mmHg on both hands and he had a 90-bpm regular pulse with a low-amplitude left femoral pulse without clinical evidence of lower-limb ischemia. The rest of the physical examination produced findings within the normal limits. An EKG revealed a normal sinus rhythm without any ST-T changes. Figure 1 presents a chest X-ray with an aspect of bronchopneumonia and possibly superimposed pulmonary edema. Emergency transthoracic echocardiography (TTE) showed an enlarged ascending aorta (45 mm) with the intimal flap present at this level, without pericardial effusion and with normal biventricular function, severe aortic regurgitation and mild mitral and tricuspid regurgitation. The blood work revealed abnormal renal function with a serum creatinine of 2.59 mg/dL at admission and severe hepatic cytolysis (AST/ALT 4058/1362 U/L), with a spontaneous INR of 1.4, in the context of normal values a day before admission to our institute. The patient presented inflammatory syndrome and his serum lactate was 2.3 mmol/L. The patient had a negative PCR test for COVID-19 with positive Cov-2IgM. No data regarding SARS-CoV-2 variants were available. We interpreted this as a recent infection with COVID-19 complicated by bronchopneumonia.

Considering the patient’s stable hemodynamic state with no pericardial effusion present, and the poor prognosis of patients with recent COVID-19 infection and cardiac surgery with cardiopulmonary bypass, the decision to postpone emergency surgery for TAAD was made and the patient was transferred to the intensive care unit. 

**Figure 2:**
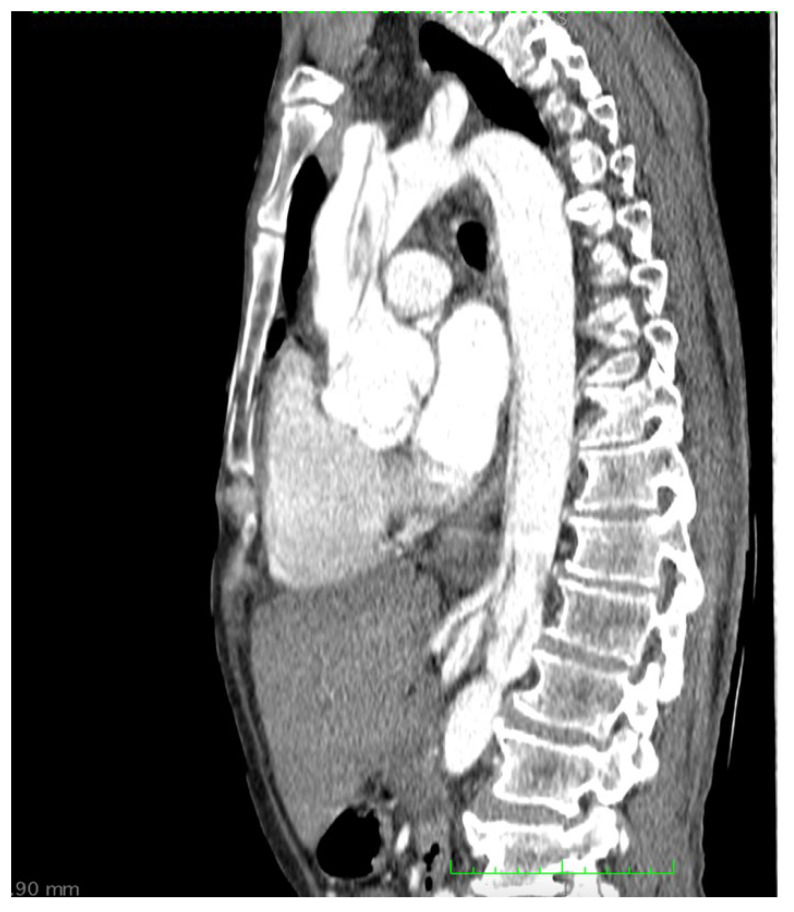
CT showing intimal flap at the level of the ascending aorta, aortic arch, thoracic and abdominal aortas and superior mesenteric artery. Celiac trunk with origin in a small true aortic lumen.

**Figure 3:**
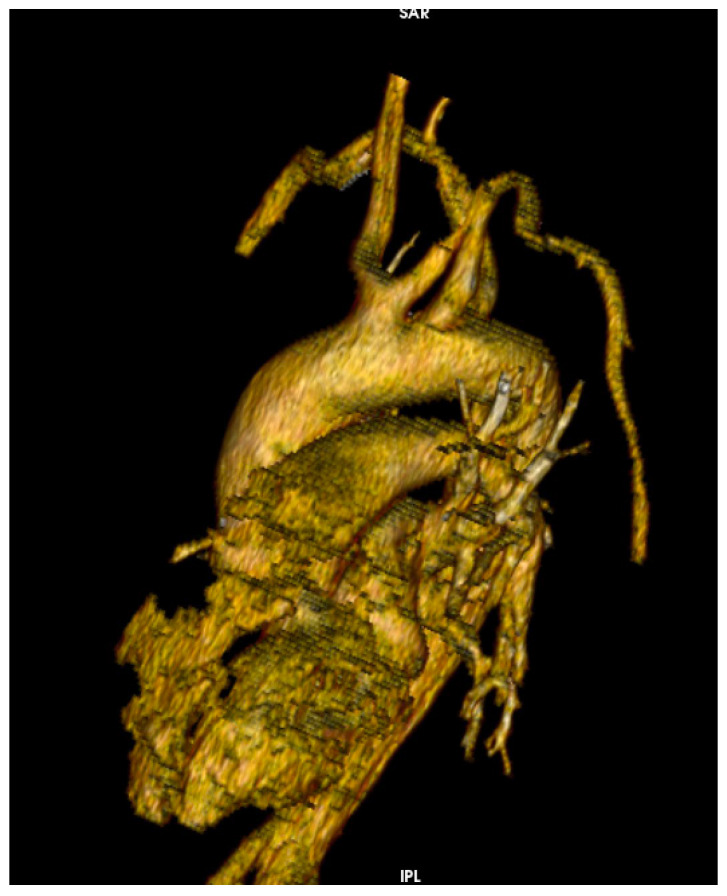
Arch anomaly—arteria lusoria and common origin of the innominate artery and left common carotid artery.

**Figure 4:**
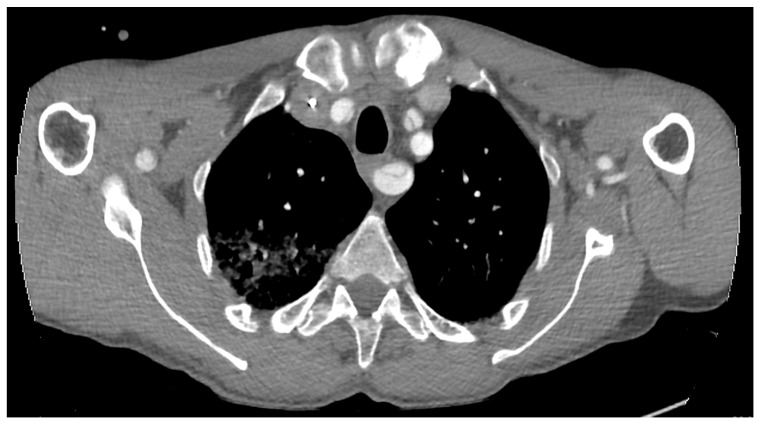
Intimal flap present in all the supra-aortic vessels.

**Figure 5:**
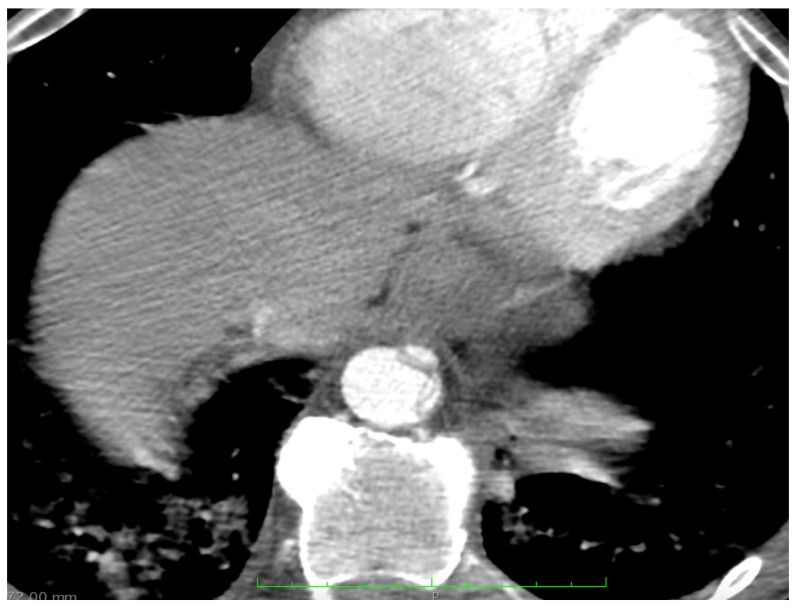
Small true lumen of the abdominal aorta at the origin of the celiac trunk, and protruding false lumen with a “double” intimal flap at this level.

**Figure 6:**
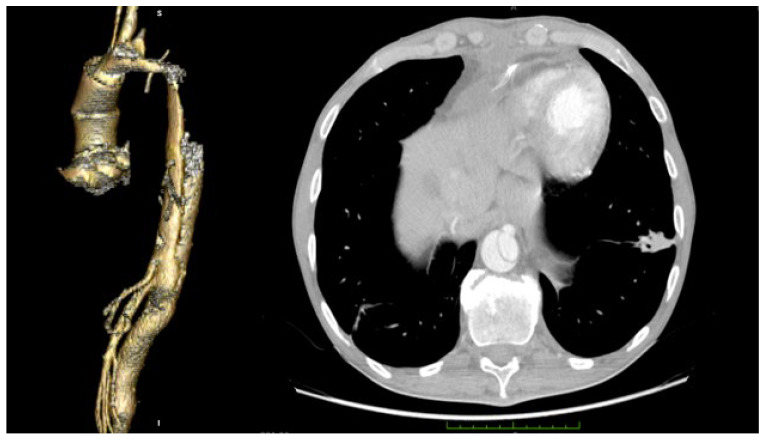
CT 3D reconstruction (**left**) and axial image (**right**) of the celiac trunk originating from the true lumen of the abdominal aorta, with an increased diameter and no compression compared to preoperative settings.

A CT scan of the brain, thorax and abdomen is presented in Figure 2. The CT scan showed an intimal flap present at the level of the ascending aorta, aortic arch and descending aorta, extending to the left external iliac artery, without any acute brain lesions. Figure 3 presents a congenital arch anomaly, with the right subclavian artery originating distal to the left subclavian artery (arteria lusoria), and a common origin for the innominate artery and left common carotid artery. Figure 4 shows the intimal flap present in all the supra-aortic vessels. Both carotid arteries and subclavian arteries presented intimal flaps. True lumen at the level of celiac trunk origin was about 3 mm. At this level, a false lumen appears to protrude near the origin of the celiac trunk, and a double intimal flap can be observed in Figure 5, suggesting a possible dynamic obstruction, consistent with the severe cytolytic syndrome at admission. Figure 6 presents a 3D CT reconstruction and axial image of the celiac trunk originating from the true lumen of the abdominal aorta, with an increased diameter and no compression compared to preoperative settings. The superior mesenteric artery was also dissected with both lumens circulated (Figure 2). Both renal arteries were circulated with no evidence of renal ischemia, the right renal artery being supplied from the false lumen.

A lung scan showed multiple, bilateral areas of consolidation with air bronchogram included and areas of ground-glass attenuation with a mainly central distribution, present in two-thirds of both pulmonary parenchyma (more significant in the right lung), suggesting an infectious pneumonia (Figure 1).

The patient was held back from undergoing central aortic surgery for 6 days. His management in the intensive care unit targeted both the bronchopneumonia and respiratory failure and blood pressure control.

Blood pressure control met a target of 100–120 mmHg systolic pressure. Initially, the patient was normotensive, but he became progressively hypertensive due to acute aortic regurgitation. This issue was addressed on day 3 with a combination of alpha-blockers, loop diuretic and calcium channel blockers.

Repeated control of serum parameters was carried out to monitor the renal and hepatic function.

Antibiotic therapy was started initially with a combination of ceftriaxone and clarithromycin. The patient required respiratory support with high-flow oxygen therapy at 50–60 L/min, with a FiO_2_ of 0.6 to maintain a blood oxygen saturation of 97–99%. The patient was afebrile by day 3, without a productive cough and with normal pulmonary sounds. By day 5, his WBC decreased from 16.340/μL to 8.220/μL, his CRP was constant at 50 mg/L and his procalcitonin decreased from 1.36 to 0.23 ng/mL. We did not consider a specific treatment for COVID-19 (antivirals, steroids) to be necessary, and no anticoagulation regimen was given because we considered the risk of aortic rupture and bleeding too important.

The hepatic and renal dysfunction began to progressively improve from day 2, the AST/ALT decreased from 4058/1362 U/L to 124/238 U/L and the serum creatinine normalized to 0.87 mg/dL with preserved diuresis. We did not consider any percutaneous reperfusion to be necessary and interpreted this as a dynamic obstruction from AD.

The serum lactate normalized on day 2. Resolution of rhabdomyolysis was observed with a CK/CK-MB of 146/29 U/L by day 5.

Serial transthoracic echocardiogram examinations showed severe aortic regurgitation with good biventricular function, mild mitral and tricuspid regurgitation and no pericardial effusion.

**Figure 7:**
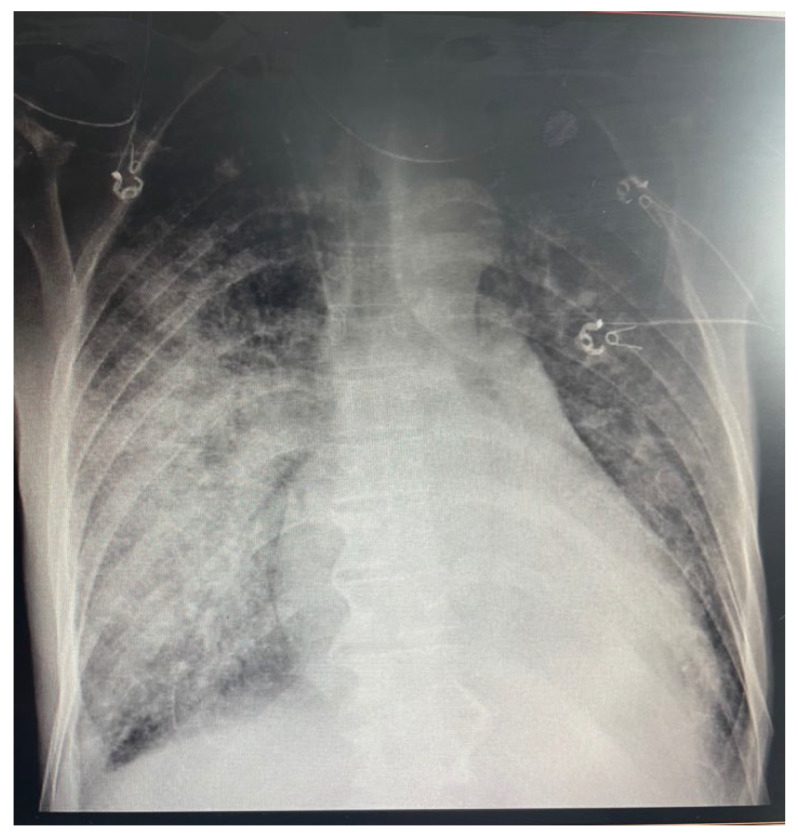
Chest XR at day 6 showing progression of pulmonary lesions suggesting superimposed pulmonary edema.

On day 6, the patient’s respiratory status worsened, with a chest XR suggesting a superimposed pulmonary edema (Figure 7). A transthoracic echocardiogram showed the progression of the mitral and tricuspid regurgitation from mild to moderate, with a 45% ejection fraction of the left ventricle and mild dysfunction of the right ventricle. Minimal pericardial effusion was present at this time. Intermittent non-invasive CPAP was initiated to maintain a blood oxygen saturation of 97–100%. The antibiotic therapy was changed to a carbapenem antibiotic (Meropenem). The decision to proceed with surgery was taken considering the risk of aortic rupture and worsening heart failure.

Surgery proceeded in the ascending aorta with hemiarch replacement, with circulatory arrest at 28 °C and selective cerebral perfusion via the innominate artery and left carotid artery. We made an intraoperative observation of a circumferential intimal tear 1 cm above the aortic commissures. No intimal tear was observed in the aortic arch or at the origin of the arch vessels. The total circulatory arrest time was 39 min, with 35 min of cerebral perfusion, and the total cardiopulmonary bypass time was 158 min. The patient did not present symptoms of arteria lusoria so we did not consider surgical correction to be necessary. Transesophageal echocardiography at the conclusion of surgery confirmed minimal aortic, mitral and tricuspid regurgitation, with a 45% left ventricular ejection fraction and normal right ventricle function.

The patient was extubated at 23 h and required a low dose of inotrope and vasopressor medication in the first 2 days postoperatively. The respiratory management consisted of inhalation therapy with colistin every 8 h for 7 days, after a positive pharyngeal exudate with pseudomonas aeruginosa MDR+ 3 days after surgery. The patient did not require any other noninvasive respiratory treatment after surgery, with SpO_2_ of 97–100% only with a facial mask. Serial chest XRs showed the resolution of the pulmonary lesions, as was confirmed with a CT scan (Figure 1). The hepatic and renal function continued to improve to normal at discharge 11 days after surgery. The postoperative chest CT showed complete thrombosis of the false lumen at the level of both common carotid arteries, subclavian arteries and aortic arch, and no progression of the intimal flap at the level of the descending aorta and branches. The celiac trunk originated from the true lumen with a diameter of 1.3 cm, significantly larger than preoperative settings. The superior mesenteric artery presented an intimal flap, with both lumens being circulated (Figure 6).

Serial TTE revealed mild aortic, mitral and tricuspid regurgitation with normal biventricular function and without pericardial effusion. The patient was discharged on the 11th postoperative day.

## 3. Discussion

Acute aortic dissection (AAD) is part of acute aortic syndrome (AAS), together with intramural hematoma and penetrating aortic ulcers [13]. Medical treatment alone is associated with 20% mortality by 24 h after presentation, 30% by 48 h, 40% by 7 days and 50% by 1 month. Mortality rates are high, even with surgical management (10% by 24 h and 20% by 30 days). The most common causes of death include aortic rupture, cardiac tamponade, stroke, visceral ischemia and circulatory failure [14].

COVID-19 has been declared a pandemic since March 2020. Although it is primarily an acute respiratory disease, extrapulmonary manifestations such as cardiovascular complications exist. This includes acute myocardial injury, arrhythmias, cardiogenic shock, acute coronary syndromes and venous thromboembolism [4]. Li et al. [15] reported an incidence of 17.1% for hypertension and 16.4% for cardio-cerebrovascular disease in patients with COVID-19, and 8% acute cardiac injury in patients with COVID-19. In patients transferred to the ICU or severe cases, the incidence of hypertension increased twofold and threefold for cardio-cerebrovascular disease, respectively, while the incidence of acute cardiac injury increased dramatically by 13-fold for both patient types. The researchers concluded that infection with COVID-19 can aggravate damage to the heart. Wang et al. [16] also reported high incidences of cardiac complications in these patients. Acute cardiac injury was present in 7.2%, shock in 8.7% and arrhythmia in 16.7% of cases, with a higher prevalence of each in patients with ICU admission. One of the possible mechanisms proposed for cardiac complications in patients with COVID-19 is the ability of SARS viruses to bind to specific receptors such as angiotensin-converting enzyme 2 (ACE2) [17]. These receptors are expressed in the heart, providing a link between coronaviruses and the cardiovascular system. It is believed that downregulating the ACE2 pathway in the lungs and in the myocardium leads to myocardial inflammation, lung edema and acute respiratory failure [4]. Moreover, the systemic inflammatory and procoagulant state persists after the resolution of initial viral infections, with an increased cardiovascular risk for up to 10 years [18].

Several studies are establishing a link between infection with COVID-19 and AD. At a molecular level, there are multiple similarities between AD and inflammation caused by COVID-19. Excessive activation of TGF-B is present in COVID-19 patients, and it is known that Marfan syndrome patients’ high levels of TGF-B are correlated with aortic root dilatation and high risk of AD [6]. Total plasma homocysteine (tHCy) is a marker of aortic sclerosis, and in Marfan syndrome patients, is correlated with the incidence of AD. In patients, with COVID-19, high levels of tHCy can be used as a predictor of the severity of the disease [19]. Plus, high expression of matrix metalloproteinases (MMPs) produces degeneration of collagen and elastin fibers that leads to aortic aneurysms in Marfan syndrome patients, with high levels of MMPs also described in patients with COVID-19 [20,21].

Cardiac surgery with cardiopulmonary bypass in the setting of COVID-19-positive patients carries a high risk of postoperative respiratory failure, with one study reporting an incidence of 56%, where more than half of the patients required invasive ventilation [22]. Another study reported that readmission to the ICU for pulmonary complications in these patients is associated with a mortality of 75% [23]. The results of urgent surgery for AD and COVID-19 infections were variable in a review of three case reports [24]. One patient died of progressive respiratory failure on day 4 after surgery, one survived after intensive management of postoperative viral and bacterial pneumonia and one patient had an uneventful recovery.

There have also been reports of increased complications during cardiopulmonary bypass. Coagulation and oxygenator gas-exchange complications were significantly higher in patients with COVID-19 [25]. While further studies are required to evaluate the possible benefits of delaying cardiac surgery in COVID-19 patients, there are some that advocate for a period of 2–4 weeks before surgery in asymptomatic patients [26].

Our patient did not present any of the classic risk factors for AD, with his past medical history significant only for the recent COVID-19 infection with superimposed bronchopneumonia at admission. He had a normal tricuspid aortic valve without ascending aortic dilatation, no history of hypertension or drug abuse and no family history of cardiovascular disease. There are recent studies that suggest that AD can be an extrapulmonary complication of COVID-19, the link between them being the hyperinflammatory state associated with the viral infection. However, the notion that infection with COVID-19 can induce an AD is only a hypothesis, and several studies are needed to confirm this.

At presentation, our patient had severe cytolytic syndrome and elevated creatinine levels, with normal values one day before. We interpreted this as visceral malperfusion syndrome caused by a dynamic obstruction, which is supported by the fact that no other percutaneous solutions were necessary over the next 6 days to manage the MS. Indeed, there are studies that show that resolution of MS occurs in 80% of cases with urgent surgery [27]. However, the association of cardiopulmonary bypass with MS in AD carries a high risk of postoperative respiratory failure because of reperfusion injury [28,29].

Considering all of the above, our patient presented two conditions that in our opinion would have led to a poor postoperative prognosis in the setting of emergency central aortic surgery: post COVID-19 bronchopneumonia with respiratory failure and a dynamic malperfusion syndrome with severe cytolytic syndrome and renal failure. Combined, these comorbidities would have created a high probability of rapidly progressive respiratory failure, and possibly, death.

## 4. Conclusions

Careful monitoring and aggressive management of this kind of patient are mandatory when the decision is made to postpone surgery. Delaying central aortic therapy in patients with TAAD must always be opted for with consideration of the imminent risk of aortic rupture, which remains the main predictor of mortality. Plus, further important factors are coronary and myocardial involvement and visceral ischemia. Follow-up of liver and renal function in our case led to the conclusion that a dynamic occlusion of the celiac trunk and right renal artery was the cause of the severe cytolytic syndrome and renal dysfunction. The need for an ultrasound to monitor the reno-visceral perfusion was indicated, together with serum parameters. We did not consider it necessary to proceed with percutaneous treatment (stenting or percutaneous fenestration) because of rapid improvement of the renal and hepatic function starting from the second day of admission. The decision to proceed with surgery after initial management of the respiratory failure and bronchopneumonia with antibiotics and noninvasive respiratory support was due to worsening of the heart failure and a high risk of aortic rupture after delaying the surgery for 6 days.

## Data Availability

Data available on request.
